# Underwhelming or Misunderstood? Genetic Variability of Pattern Recognition Receptors in Immune Responses and Resistance to *Mycobacterium tuberculosis*

**DOI:** 10.3389/fimmu.2021.714808

**Published:** 2021-06-30

**Authors:** Jean-Yves Dubé, Vinicius M. Fava, Erwin Schurr, Marcel A. Behr

**Affiliations:** ^1^ Department of Microbiology and Immunology, McGill University, Montreal, QC, Canada; ^2^ Program in Infectious Diseases and Immunity in Global Health, The Research Institute of the McGill University Health Centre, Montreal, QC, Canada; ^3^ McGill International TB Centre, McGill University, Montreal, QC, Canada; ^4^ Department of Human Genetics, Faculty of Medicine, McGill University, Montreal, QC, Canada; ^5^ Department of Medicine, Faculty of Medicine, McGill University, Montreal, QC, Canada

**Keywords:** mycobacterium tuberculosis, tuberculosis, pattern recognition receptor (PRR), genetic association studies (GAS), C-type lectin receptors (CLRs), NOD-like receptors (NLRs), toll-like receptors (TLR), microbe associated molecular pattern (MAMP)

## Abstract

Human genetic control is thought to affect a considerable part of the outcome of infection with *Mycobacterium tuberculosis* (*Mtb*). Most of us deal with the pathogen by containment (associated with clinical “latency”) or sterilization, but tragically millions each year do not. After decades of studies on host genetic susceptibility to *Mtb* infection, genetic variation has been discovered to play a role in tuberculous immunoreactivity and tuberculosis (TB) disease. Genes encoding pattern recognition receptors (PRRs) enable a consistent, molecularly direct interaction between humans and *Mtb* which suggests the potential for co-evolution. In this review, we explore the roles ascribed to PRRs during *Mtb* infection and ask whether such a longstanding and intimate interface between our immune system and this pathogen plays a critical role in determining the outcome of *Mtb* infection. The scientific evidence to date suggests that PRR variation is clearly implicated in altered immunity to *Mtb* but has a more subtle role in limiting the pathogen and pathogenesis. In contrast to ‘effectors’ like IFN-γ, IL-12, Nitric Oxide and TNF that are critical for *Mtb* control, ‘sensors’ like PRRs are less critical for the outcome of *Mtb* infection. This is potentially due to redundancy of the numerous PRRs in the innate arsenal, such that *Mtb* rarely goes unnoticed. Genetic association studies investigating PRRs during *Mtb* infection should therefore be designed to investigate endophenotypes of infection – such as immunological or clinical variation – rather than just TB disease, if we hope to understand the molecular interface between innate immunity and *Mtb*.

## Introduction

Tuberculosis (TB) was the number one cause of death due to a single infectious agent, *Mycobacterium tuberculosis* (*Mtb*), in the year 2019 according to the WHO. SARS-CoV-2 has surpassed *Mtb* in the last year; however, deployment of vaccines and experience with containment measures should blunt the death rate from COVID-19 in the years to come, such that TB may reprise its role as the most important cause of infectious mortality. Near 40 million people have died from TB in the last 20 years while treatment has saved 60 million (WHO). Yet, in the same interval, an estimated 10- to 20-fold more people were infected but did not progress to disease (1, 2). Together, this suggests broad host control or tolerance of this pathogen, despite the important minority who progress to disease each year.

Our time together with *Mtb* has potentially spurred human adaptation to allow us as a population to subsist with this obligate pathogen. *Mtb* has been evolving to parasitize humans for millennia and within that time the relationship has possibly changed us too, when and where *Mtb* was endemic (3–5). Current and past abundance of human genetic diversity allows researchers to test the importance of genetic variation in *Mtb* infection outcomes and infer an evolutionary response by our species to survive the *Mtb* pandemic. One example where *Mtb* has potentially exerted a purifying selection on humans is that of the *TYK2* P1104A variant, which was calculated to have decreased in western Europeans concomitant with endemic TB over the last two millennia (6, 7). The *TYK2* P1104A variant is known to disrupt IL-23-dependent IFN-γ production (6) and was associated with a 5-fold increased risk for developing TB in the contemporary UK biobank (8). We are not aware of any evidence of positive selection of a TB resistance gene to date.

Is every case of TB a situation where the host genetic combination is vulnerable to *Mtb*? We can hypothesize a genetic combination impervious to *Mtb*. We may not have to extend our imagination very far, as there are documented cases of people who remain TST negative in high-burden settings, such that it is statistically unlikely that they have never inhaled *Mtb* [recently reviewed in (9)]. Therefore, developing TB is, in part, a result of genetics, and not just being a human exposed to *Mtb*, a postulate supported by the 21% heritability estimate for household contacts in Peru progressing from TST positivity to TB (10). Environmental parameters can also have an effect (e.g. level of exposure, lung damage, HIV co-infection) and thus in theory identical twins could have different outcomes with *Mtb* infection. *Mtb* also has variation which might contribute to a different outcome for the bacterium and the host: there are 9 lineages described to date (11–13) with some being deemed more virulent in experimental models (14).

Genetic variation creates differences that can fine tune a host-pathogen interaction, or abrogate it completely, resulting in altered immunity. One modality where there is a direct opportunity for co-evolution is in physical interactions between host molecules and *Mtb* molecules. These interactions can be placed into a few camps including: 1) between classical T-cell receptors and MHC molecules presenting microbial epitopes (15); 2) between antibodies and cognate microbial ligands (16); 3) between donor-unrestricted T cells and their respective mycobacterial epitopes presented on invariant host molecules operating analogously to MHC (17, 18); 4) between inborn sensors of microbial products, otherwise known as pattern-recognition receptors (PRRs), and their cognate microbe-associated molecular patterns (MAMPs). By their nature as structural molecules, MAMPs are subjected to a stronger purifying selection than many proteins. Unlike T-cell receptors, PRRs cannot generate diversity within an individual, yet there is variability amongst human population PRR gene pools as discussed further below. Most of all, should we even expect strong selective pressure on host PRRs to recognize *Mtb* MAMPs? In this paper, we sought to review what is known about the relative importance of the MAMP-PRR interaction for the mammalian host during *Mtb* infection primarily through two sources of data: 1) controlled animal experiments using engineered genetic knockouts (KOs) of PRRs; 2) natural experiments in humans where genetic diversity permits us to seek associations between polymorphisms and the course of *Mtb* infection. We later place this in perspective with genes known to have strong effects on animal outcomes and lastly discuss how to approach human genetic studies of PRRs in the years to come.

## PRRs and Their Functions Against *Mtb* at the Cellular Level

**Figure 1:**
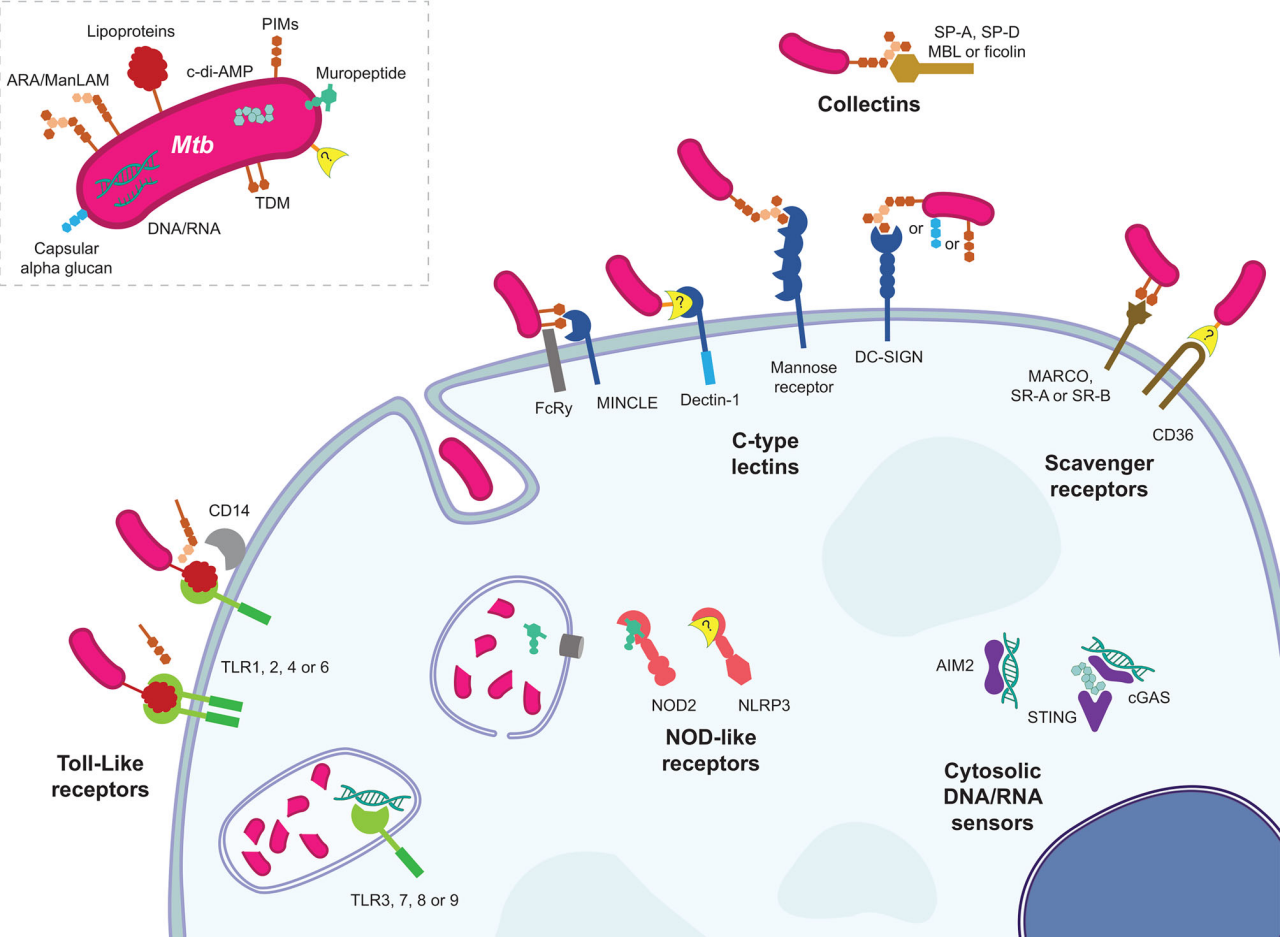
The various host PRR and *Mtb* MAMP interactions. Representative MAMP-PRR interaction are depicted in their approximate cellular locations highlighting the numerous ways in which *Mtb* announces its arrival to a phagocyte.

The interactions between many PRRs, *Mtb* and *Mtb* MAMPs have been described over the last few decades and are summarized in **Figure 1**. Various mechanisms have been uncovered by which PRR recognition of *Mtb* leads to a cellular effect. Immediately below, we briefly review the molecular functionality of the PRRs which have been demonstrated to mediate an immune response to mycobacteria. Whether these molecular and/or cellular effects translate to protection or pathology in the whole animal is examined in the subsequent section.

### Toll-Like Receptors

Toll-like receptors (TLRs) were the prototypical PRR fitting the hypothesis proposed earlier by Janeway Jr (19) that there existed inborn sensors in animals for products common among groups of microbes but absent from the host, allowing host recognition of non-self invading microbes – a form of antibody or T-cell receptor for innate immunity. The discoveries in the 1990s on the Toll gene of *Drosophila*, followed by work exploiting mutant forms of TLR4 in human cells and mice demonstrated that mammalian TLR4 was a sensor of Gram-negative endotoxin (a.k.a. lipopolysaccharide, LPS) (20–23). In total there are 10 TLRs in humans (13 in mice), each with different microbial ligands and slightly varying effects. TLR2 cooperates with TLR1 or TLR6, as well as other PRRs like CD14, to sense mycobacterial lipoproteins and lipoglycans. Identified mycobacterial TLR2 ligands include LAM (non-capped araLAM and not ManLAM) (24, 25), 19 kDa lipoprotein (LpqH), 38 kDa lipoprotein (PstS1) (26), PIMs (with differing activities) (27, 28), 27 kDa lipoprotein (LprG) (29), and LprA (30) to name a few (31). Mycobacteria including *Mtb* shed membrane vesicles containing TLR2 ligands that are sufficient to generate a TLR2-dependent immune response (32). More recently *Mtb* sulfoglycolipids have been shown to be competitive TLR2 antagonists (33). TLR4’s mycobacterial ligands are less clear, but *Mtb* extracts have TLR4-dependent stimulatory activity; many proteins have been proposed as TLR4 agonists, with GroEL1 and 2 being examples (34). TLR5, which recognizes flagellin, does not have a known mycobacterial ligand (mycobacteria do not swim – they float). TLR3, TLR7 and TLR8 recognize RNA, and recent reports revealed that they may respond to host and/or mycobacterial RNA during infection (35–37). TLR9 recognizes CG-rich DNA (i.e. CpG motifs) and has been shown to contribute to the cellular response to *Mtb*’s CG-rich genomic DNA (38, 39). TLR10 has no known ligands, mycobacterial or otherwise. Murine TLR11, 12, and 13 are not reviewed here because they have no direct relevance to human health.

A complete review of TLR signalling, not specific to mycobacteria, has been recently published elsewhere (40). Briefly, when TLRs are engaged and oligomerize on the membrane, adaptor proteins MyD88 or TRIF are recruited to the cytoplasmic side to form ‘myddosomes’ or ‘triffosomes’, respectively. These supramolecular platforms direct signalling events that lead to activation of MAPK and NF-κB pathways, for example. Such signalling begins an inflammatory response by the cell which includes upregulation of costimulatory molecules and antigen presentation by MHC molecules, plus secretion of soluble factors like cytokines, in the cases of macrophages and dendritic cells (DCs).

### C-Type Lectin Receptors

C-type lectin receptors (CLRs) are a large, diverse category of receptors of which some members function as PRRs by binding to MAMPs; other CLRs bind endogenous ligands or non-microbial exogenous ligands. The etymology of the name originates from some members requiring calcium (Ca^++^, hence “C”) to bind their respective carbohydrate ligands (hence “lectin”). There are both membrane-bound and soluble forms of CLRs. A full review of this complex category of PRRs has recently been published and provides more mechanistic detail than is presented here (41).

The mannose receptor (CD206) expressed on macrophages was shown to assist these phagocytes in uptake of the tubercule bacillus (42) with ManLAM being the mycobacterial ligand for CD206 (43). The ManLAM-CD206 interaction was later demonstrated to uniquely induce IL-8 and cyclooxengenase expression *via* PPARγ, while PPARγ knockdown was associated with reduced bacterial growth and increased TNF production during monocyte-derived macrophage infection (44). *Pparg*-KO mice had about half the pulmonary bacterial burden and reduced lung pathology than WT counterparts when aerosol infected with *Mtb* (45).

The CLR called DC-SIGN (Dendritic Cell-Specific Intercellular adhesion molecule-3-Grabbing Non-integrin, a.k.a. CD209) is a main receptor on DCs for binding to *Mtb* (46). DC-SIGN expressed on dendritic cells has been show to interact with ManLAM (47), PIM6 (48) and capsular alpha-glucan (49). The DC-SIGN homologues L-SIGN (human) and SIGNR1 (mouse, one of five homologues), have been shown to interact with ManLAM too (50). DC-SIGN ManLAM ligation modulated TLR-induced signalling (e.g. NF-κB pathway) *via* Raf-1 (51, 52).

MINCLE (Macrophage inducible Ca^++^-dependent lectin receptor, encoded by *CLEC4E*) associates with FcRγ to bind mycobacterial cord factor trehalose-6,6’-dimycolate (TDM) and TDM is sufficient to induce granuloma formation in murine lungs if functional MINCLE and FcRγ are present (53). MINCLE signals *via* the SYK-CARD9 pathway to lead to the production of proinflammatory cytokines (54). *Card9*-KO mice succumb more quickly to *Mtb* than WT, associated with defective anti-inflammatory signalling presumably leading to lethal immunopathology (55). MINCLE expression is low in resting macrophages, and first requires induction *via* signalling through MCL (encoded by *Clec4d* and not to be confused with MCL-1). MCL is also a FcRγ-coupled and TDM CLR but cannot mediate the same pro-inflammatory response on its own (56). MINCLE and MCL expression are co-dependent (57, 58).

Dectin-1 was shown to mediate part of the immune response of splenic DCs to *Mtb* (59). *Mif*-KO mice have impaired survival and immunity compared to WT during aerosol infection with *Mtb* HN878 strain, while bacterial killing and cytokine production were nearly restored when *Mif*-KO cells were complemented with Dectin-1 (overexpressed). These results suggest the MIF defect mostly manifests in defective Dectin-1 signalling (60). The mycobacterial ligand for Dectin-1 remains unknown. Dectin-2 was reported to recognize ManLAM (61, 62), but pathogenesis studies have yet to be done with this CLR.

Recently, DCAR (dendritic cell immunoactivating receptor; encoded by *Clec4b1*), also a FcRγ-coupled CLR, was demonstrated to be a receptor of PIMs. DCAR is expressed on monocytes and macrophages. *Clec4b1*-KO mice had partially defective immune responses and bacterial control during BCG infection (63).

### Soluble CLRs

Collectins are soluble, non-cell-bound proteins; they are CLRs in that they specifically associate with sugars on the surface of microbes to mediate an effect. Surfactant proteins (SP) are collectins that exist in pulmonary surfactant. SP-A promoted attachment and phagocytosis of *Mtb* by alveolar macrophages by a mechanism that required mannose receptor but not SP-A contacting *Mtb* (64, 65). SP-A supressed nitrite production from AMs preventing *Mtb* killing and controlling bacterial growth (66). SP-A was shown to bind to ManLAM (67) and APA, the alanine- and proline-rich antigenic glycoprotein (68). SP-D binds to ManLAM and agglutinates *Mtb*, but in contrast to SP-A, SP-D reduced *Mtb* binding to macrophages (69). Reduced uptake occurred without agglutination using a modified SP-D (70). However, SP-D increased phagosome-lysosome fusion, but did not alter the respiratory burst (71).

The collectin mannan-binding lectin (MBL) and ficolins are serum-borne receptors that bind to microbes to initiate the complement cascade. MBL was first demonstrated to interact with *Mtb* and *M. leprae* sonicate (72). Ficolins, of which there are at least three in humans and two in mice, are also part of the lectin-complement system. Ficolin-2 was shown to bind to *Mtb* to play a protective role involving opsonization and inflammatory signalling in macrophages (73). Another group suggested ficolin-3 was important for agglutination and phagocytosis of *Mtb* (74). MBL and ficolins were suggested to bind to ManLAM and/or Ag85 (75, 76).

### NOD-Like Receptors

Nucleotide-binding Oligomerization Domain-containing (NOD)-like receptors (NLRs) are a group of cytoplasmic sensors. Reviews with more detail on their mechanisms of action than presented here have been published, for example (77). Its members NOD1 and NOD2 are essential in the detection of peptidoglycan fragments D-glutamyl-meso-diaminopimelic acid (iE-DAP) and muramyl dipeptide (MDP), respectively (78–81). Both NOD1 and NOD2 signal through the adaptor protein RIPK2 to reach NF-κB and MAPK pathways. Few reports have been published on an important role for NOD1 during *Mtb* infection, one showing NOD1 plays a role in cytokine production only in the absence of NOD2 or after LPS-pretreatment of BMDMs (82). Mycobacteria do possess the iE-DAP moiety in their peptidoglycan (83). In contrast, NOD2 has been well-studied in *Mtb* infection [Pubmed searches of “Mycobacterium tuberculosis AND NOD1” or “…NOD2” yielded 7 and 57 hits, respectively, at the time of writing (15-2-2021)].

Mycobacteria produce a distinct NOD2 ligand, *N*-glycolyl MDP, while most other bacteria produce *N*-acetyl MDP (84, 85). *N*-glycolylated peptidoglycan and MDP were shown to be better inducers of immune responses compared to the *N*-acetylated forms by comparing with mycobacterial KOs and synthetic MDPs (86–88). The absence of NOD2 during *Mtb* infection was accompanied by reduced nitric oxide and cytokine production from mouse macrophages (89, 90) and reduced iNOS from human macrophages (91). NOD2 signalling has been called “non-redundant” in that although there are shared pathways with TLR and CLR signalling (e.g. NF-κB), NOD2 signalling appears to work synergistically with other MAMPs and little immune response is produced with MDP stimulation alone (86, 88, 92).

Only one other NLR has been significantly studied in the context of *Mtb* infection: NLRP3 (NLR family pyrin domain containing 3). There is no known mycobacterial ligand for this NLR – it has been suggested that NLRP3 can sense specific host products in the context of infection (77). Recently, ESX-1-mediated membrane damage has been tied to caspase-1 activation, NLRP3 oligomerization, inflammasome formation and finally IL-1β release (93).

### Nucleic Acid Cytosolic Surveillance Receptors

AIM2 (absent in melanoma 2) appears to play a role in *Mtb* infection. This cytosolic DNA receptor was necessary for full caspase-1 cleavage/activation and IL-1β release during *Mtb* infection and *Mtb* DNA transfection (94). STING (Stimulator of interferon genes), part of a cytosolic DNA sensing system, was essential for autophagy targeting of ESAT-6-producing mycobacteria in mouse bone marrow-derived macrophages (95) and zebrafish embryos (96). cGAS (cyclic guanosine monophosphate–adenosine monophosphate (cGAMP) synthase), a DNA sensor that works with STING, was required for *Mtb* autophagy in addition to STING (97). Type I IFN production during *Mtb* infection elicited by cGAS-produced, STING-sensed cGAMP was dependent on RD-1 (98, 99). *Mtb*-produced c-di-AMP was also shown to contribute to type I IFN production through STING (100). It has been suggested that *Mtb* DNA engagement of the AIM2 inflammasome leads to the inhibition of host cell-protective STING functions (101).

### Scavenger Receptors and Complement

Scavenger receptors (SR) are a diverse and poorly defined group of cell surface receptors that interact with endogenous and microbial ligands. Details of these receptors have been recently reviewed elsewhere (102, 103). Inhibitors of scavenger receptors reduced *Mtb* binding to macrophages (104).

MARCO is a scavenger receptor that was suggested to bind and “tether” *Mtb* to a macrophage’s surface by interacting with TDM (105), and zebrafish lacking MARCO expression had reduced macrophage uptake of *M. marinum* (106). Similarly, blocking MARCO on human mesenchymal stems cells reduced *Mtb* uptake (107).

KO of the gene encoding scavenger receptor A (SR-A) did not affect inflammatory gene transcription during *Mtb* infection (108). The KO increased TNF and MIP-1α production from AMs treated with TDM (109). Overexpression of Scavenger receptor B1 (SR-B1) in immortal cells was associated with increased BCG and *Mtb* binding to the cells, but the corresponding KO in murine macrophages had no effect on BCG binding (110). SR-B1 was essential for EsxA-mediated transcytosis of *Mtb* across M cells (111). A related SR-B family member, CD36, was identified with a *Drosophila* RNAi screen to be essential for uptake of *M*. *fortuitum* (112). CD36 knockdown in human monocyte-derived macrophages reduced surfactant lipid uptake as well as intracellular growth of *Mtb*, suggesting CD36 normally promotes intracellular *Mtb* growth or survival (113). Another group showed *Cd36* KO macrophages control *Mtb*, BCG and *M. marinum* infections better, independent of phagocytosis rate, nitric oxide and ROS production. Similarly, mice receiving BCG i.p. had lower bacterial loads with *Cd36* KO *vs* WT (114). Homologues of SR-BII and CD36 in *Dictyostelium discoideum* (a social amoeba) are similarly involved in phagocytosis of *M. marinum* (115).

## Consequences of PRR KOs in Mice

The overall importance of individual genes during *Mtb* infection is best addressed in two ways: observing what happens to individuals with diverse expression or functionality of the gene of interest who perchance become infected with *Mtb* (the natural experiment); alternatively, individuals of known or controlled gene status can be intentionally infected with *Mtb* – unethical in humans and therefore animal models are necessary. As mentioned above, many PRRs are important for specific cellular processes relevant to *Mtb* infection. It is therefore hypothesized that in the absence of a PRR, certain aspects of the host-*Mtb* interaction are lost, which should result in a phenotype in the whole animal. We have also assumed that the animal would suffer most from the aberrant immune response. This assumption is perhaps too simplistic: *Mtb* is a professional, obligate pathogen, and although it is perceived as hard-to-kill, it might also suffer from a host environment that does not behave as *Mtb* has evolved to ‘expect’. Additionally, we have suggested that the potential coevolution of humans and *Mtb* has shaped the PRR-MAMP interaction, which clearly would not apply in infections of animals like mice, which are not natural hosts for *Mtb*. However, one can still use mice to generate testable hypothesis for human studies and validate genetic effects observed first in humans. Numerous KO mouse studies of *Mtb* infection have been performed over the years with hypotheses of defective immunity in the animal that should manifest as decreased survival, increased bacterial burden and/or detectable differences in the immune response (e.g. bronchoalveolar lavage cytokines or T-cell defects).

### Systematic Literature Search of *Mtb* and PRRs

To non-biasedly form a conclusion as to the importance of PRRs during *Mtb* infection in animal models, we used the Medline database *via* Pubmed to repeatedly search every known PRR and its role in *Mtb* infection in a living animal with the term below (where “[PRR]” was changed in each search):

“Mycobacterium tuberculosis AND [PRR]”

Where PRR names were ambiguous, we searched multiple times using the different names. After removing duplicates, this search produced over 1100 papers, which were screened for data using KO animals during *Mtb* infection. The results of this *in silico* exercise are summarized in **Table 1**. We have added a few studies of which we were aware but that were missed by the screen (noted in **Table 1**). It is possible that other appropriate data are absent; however the non-biased approach reinforces the validity of our subsequent conclusions.

**Table 1:** Results of KO mouse studies in *Mtb* infection.

PRR KO(S)	DOSE, CFU	STRAIN	ROUTE	Δ SURVIVAL^A^	Δ MTB^B^	Δ IMM^C^	NOTES^D^	SOURCE
***TLR2***	100	H37Rv	aero	-	N	N		Reiling et al. (116)
	2,000	H37Rv	aero	Y (60/150)	–	Y		Reiling et al. (116)
	100	Kurono	aero	-	Y (1)	Y		Sugawara et al. (117)
	100	H37Rv	aero	N	Y (1)	–		Drennan et al. (118)
	500	H37Rv	aero	Y (100/>155)	Y (1)	Y		Drennan et al. (118)
	75	H37Rv	aero	N	N	N	manual	Bafica et al. (119)
	20	H37Rv	aero	-	Y (<1)	Y		Tjärnlund et al. (120)
	100	H37Rv	aero	–	N	–		Hölscher et al. (121)
	100,000	H37Rv	i.t.	-	Y (1)	Y		Carlos et al. (122)
	150	H37Rv	i.n.	–	–	Y		Teixeira-Coelho et al. (123)
	10,000,000	H37Rv	i.v.	-	-	Y		Choi et al. (124)
	75	Erdman	aero	–	Y (1)	–		McBride et al. (125)
	10	Erdman	aero	-	Y (2)	-		McBride et al. (126)
	100	Erdman	aero	–	Y (2)	Y		McBride et al. (126)
	150	Erdman	aero	-	Y (1)	-		McBride et al. (126)
	100	Erdman	aero	–	Y (<1)	Y	chimera	Konowich et al. (127)
	20	HN878	aero		Y (3)	Y		Gopalakrishnan et al. (128)
***RP105***	200	H37Rv	aero	–	Y (<1)	Y		Blumenthal et al. (129)
***TLR4***	100	H37Rv	aero	Y (180/>250)	Y (1)	Y	HeJ/HeN	Abel et al. (130)
	100	H37Rv	aero	–	N	N	HeJ/HeN	Reiling et al. (116)
	2,000	H37Rv	aero	N	-	N	HeJ/HeN	Reiling et al. (116)
	144	Erdman	aero	N	N	–	HeJ/other C3H	Kamath et al. (131)
	472	Erdman	aero	N	N	N	HeJ/other C3H	Kamath et al. (131)
	75	H37Rv	aero	N	Y (-1)	N	HeJ/OuJ	Shim et al. (132)
	100,000	H37Rv	i.n.	Y (90/>110)	Y (<1)	Y	HeJ/HeN	Branger et al. (133)
	500,000	H37Rv	i.n.	N	–	–	HeJ/HeN	Branger et al. (133)
	20	H37Rv	aero	-	Y (<1)	Y		Tjärnlund et al. (120)
	100	H37Rv	aero	–	N	–		Hölscher et al. (121)
	150	K strain	aero	-	Y (2)	Y	HeJ/HeN and B6	Park et al. (134)
***CD14***	100	H37Rv	aero	–	N	N		Reiling et al. (116)
	100,000	H37Rv	i.n.	Y (>224/210)	N	Y		Wieland et al. (135)
***LBP***	100,000	H37Rv	i.n.	N	N	Y		Branger et al. (136)
***TLR6***	100	Kurono	aero	-	N	N		Sugawara et al. (117)
***TLR9***	75	H37Rv	aero	Y (90/>150)	N	Y	manual	Bafica et al. (119)
	500	H37Rv	aero	Y (45/>90)	Y (<1)	Y (not shown)	manual	Bafica et al. (119)
	100	H37Rv	aero	–	N	–		Hölscher et al. (121)
	75	Erdman	aero	-	N	N		Gopalakrishnan et al. (137)
***SIGNR1***	100,000	H37Rv	i.n.	–	N	Y		Wieland et al. (138)
	1,000	H37Rv	i.n.	-	N	-		Tanne et al. (139)
	200	H37Rv	i.n.	–	Y (score)	Y		Court et al. (140)
***SIGNR3***	1,000	H37Rv	i.n.	-	Y (1)	Y		Tanne et al. (139)
***SIGNR5***	1,000	H37Rv	i.n.	–	N	–		Tanne et al. (139)
***CD206(MR)***	200	H37Rv	i.n.	-	N	N		Court et al. (140)
***MINCLE***	100	H37Rv	aero	–	Y (>-1)	N		Heitmann et al. (141)
	100	Erdman	aero	-	Y (<1)	Y		Lee et al. (142)
***MCL***	100	H37Rv	aero	Y	Y (<1)	Y	N>30 for survival	Wilson et al. (143)
***DECTIN-1***	100	H37Rv	aero	N	Y, (>-1)	N		Marakalala et al. (144)
***FICOLIN-A/2***	1,000,000	H37Rv	i.v.	Y (10/22)	–	–		Luo et al. (73)
***SP-A***	50	Erdman	aero	-	N	Y		Lemos et al. (145)
	6,000	Erdman	aero	–	Y (<1)	–		Lemos et al. (145)
***SP-D***	50	Erdman	aero	-	N	Y		Lemos et al. (145)
	6,000	Erdman	aero	–	Y (<1)	–		Lemos et al. (145)
***NOD2***	35	1254	aero	-	N	N		Gandotra et al. (89)
	1,500	H37Rv	aero	–	N	N		Gandotra et al. (89)
	400	H37Rv	aero	Y (200/>230)	Y (<1)	Y		Divangahi et al. (90)
***NLRP3***	300	H37Rv	aero	N	N	Y		McElvania TeKippe et al. (146)
	100	H37Rv	aero	-	N	Y		Walter et al. (147)
	300	H37Rv	aero	–	N	N		Dorhoi et al. (148)
***NLRP12***	300	H37Rv	aero	N	N	N	manual	Allen et al. (149)
***NLRC3***	200	H37Rv	aero	–	Y (-1)	Y	manual	Hu et al. (150)
***NLRC4***	300	H37Rv	aero	N	-	-		McElvania TeKippe et al. (146)
***cGAS***	200	Erdman	aero	Y (150/210)	N	–		Collins et al. (97)
	100	Erdman	aero	N (100)	N	Y	manual	Watson et al. (99)
	1,000	H37Rv	i.n.	N	N	N		Marinho et al. (151)
***STING***	200	Erdman	aero	N	N	-	gt/gt STING	Collins et al. (97)
	1,000	H37Rv	i.n.	N	N	N		Marinho et al. (151)
***AIM2***	1,000,000	H37Rv	i.t.	Y (45/>56)	Y (1)	Y		Saiga et al. (94)
***Marco***	200	H37Rv	i.n.	–	Y (score)	Y		Court et al. (140)
***SR-A***	200	H37Rv	i.n.	-	N	N		Court et al. (140)
	75	H37Rv	aero	Y (>430/230)	Y (-1, ns)	Y		Sever-Chroneos et al. (152)
***SR-B1***	100	H37Rv	aero	-	N	N		Schafer et al. (110)
	1,000	H37Rv	aero	–	N	Y		Schafer et al. (110)
***CD11b(CR3)***	200,000	Erdman	i.v.	N	N	-	3 backgrounds	Hu et al. (153)
	100,000	Erdman	i.v.	–	Y (<1, ns)	–		Melo et al. (154)
***TLR-2/4***	60	H37Rv	aero	-	N	N		Shi et al. (108)
	600	H37Rv	aero	–	N	N		Shi et al. (108)
	100	H37Rv	aero	-	N	-		Hölscher et al. (121)
***TLR2/4/9***	100	H37Rv	aero	–	N	N		Hölscher et al. (121)
***TLR2/9***	75	H37Rv	aero	Y (90/>150)	Y (<1)	Y	manual	Bafica et al. (119)
	75	H37Rv	aero	Y (120/>280)	–	–	manual	Mayer-Barber et al. (155)
	75	Erdman	aero	-	N	N		Gopalakrishnan et al. (137)
***NOD2/TLR2***	100	H37Rv	aero	–	N	–		Gandotra et al. (89)
***CD206/SIGNR1***	200	H37Rv	i.n.	-	N	N		Court et al. (140)
***SR-A/CD36***	200	H37Rv	i.n.	–	N	N		Court et al. (140)
***SP-A/D***	50	Erdman	aero	-	N	Y		Lemos et al. (145)
	6000	Erdman	aero	–	Y (<1)	–		Lemos et al. (145)

A, change in survival (Yes/No) (median survival KO/median survival control). B, change in pulmonary Mtb CFU burden (Yes/No) (maximum log KO/control). C, change in immune response observed (Yes/No). D, any irregularities from other studies (manual means source was not found in systematic search and was added manually afterwards).

In **Table 1**, we summarized the results of individual experiments presented in the literature on murine *Mtb* infections comparing a PRR KO to the ‘WT’ control animal. All data found were exclusive to the mouse. We have included the dose, *Mtb* strain and route of infection per experiment. *Mtb* can establish an infection *via* the lungs with just a few bacilli (156, 157), and therefore models using large doses and atypical routes may represent different aspects of *Mtb* disease but not necessarily follow the normal mode of infection. The magnitude of disease in mice also changes with the strain of *Mtb*, where for example the H37Rv strain is expected to be less virulent than the related Erdman strain and the HN878 strain. It is possible that some of the different outcomes across different studies addressing the same PRR knockout were due to differences in the infection model. However, our review of the data did not reveal an obvious effect of dose, strain nor route (**Table 1**).

Where survival data were present, it was clear that PRRs can have an effect on survival, although in most cases there was either no significant difference in survival from WT to KO, or it was quantitatively small. There were two instances where KO mice survived longer than WT [CD14 (135) and SR-A (152)], demonstrating that some host systems are detrimental to *Mtb* tolerance.

### TLR KOs Resulted in Small and Inconsistent Effects on Survival and *Mtb* Burden

For TLR2, two of four experiments showed reduced survival in KOs. A single *Tlr6*-KO study did not show a difference in bacterial burden nor immune response (117). For TLR4, two of seven experiments showed reduced survival in KOs. Note that many TLR4 studies took advantage of the C3H/HeJ mouse (having a spontaneous *Tlr4* loss-of-function mutation) employing other only somewhat related C3H strains as wildtype control. The maximum difference in pulmonary bacterial burden observed in most of these papers was approximately one log more in *Tlr2* or *Tlr4* KOs *vs* WT. Defects in immune responses were observed in a majority of *Tlr2* KO experiments and a minority of *Tlr4* KO experiments. Two experiments with *Tlr9* KO from one study showed more rapid death with high dose infection compared to low dose, and only the high-dose resulted in a statistically significant increase in pulmonary bacterial burden (158). No survival data for other TLRs have been published. Interestingly, most experiments with combination KOs of *Tlr2*, *4* and/or *9* resulted in no differences in bacterial burden nor immunologic responses. Two of seven experiments (*Tlr2/9* double KOs) resulted in shortened survival times, but with small or unreported differences in bacterial burden. Together, mutations in TLRs, even multiple, had only modest or negligible effects on the host’s survival and bacterial control but were frequently associated with altered immune responses. In particular, TLR2 and 9 stood out.

Of note, *Myd88*-KO mice succumbed rapidly (all dead within 1-2 months) to *Mtb* infection, despite TLRs seeming to be largely dispensable. This was attributed to the necessity of MyD88 for IL-1R signalling (*Il1r1* KO mice are equally susceptible) and intrinsic macrophage function requiring MyD88 (121, 159, 160). An earlier report with *Myd88*-KO mice showed a nearly 2-log increase in pulmonary colony-forming units (CFU) compared to WT but mice survived at least 12 weeks with limited immunological changes; no survival was presented (161).

### Few CLR KOs Resulted in Small Reductions in Survival and Bacterial Control

For CLRs, only MCL and Dectin-1 were found by us to have been disrupted in *Mtb* survival challenges. In one report, MCL (*Clec4d*)-KO caused a significant difference in survival, but specifically this was 20% mortality by 6 weeks, after which no *Clec4d*-KO mice died to week 10 (when the experiment was ended) (143). However, in the same study, pulmonary bacterial burden was less than half a log higher in the KO at four months (no significant difference at 2 months). Proinflammatory immunologic responses were elevated in the KO. Thus, MCL might play a role early in infection to control the immune response, but not so much for bacterial control.

Another lone report showed Dectin-1 (*Clec7a*)-KO mice did not have changed mortality after infection with *Mtb*, and in fact had slightly lower bacterial burdens compared to WT at 2 and 4 months post infection (144). Therefore, Dectin-1 is likely not required by the host during *Mtb* infection. No survival data was found for murine DC-SIGN homologues, mannose receptor, nor MINCLE. Only one of three studies reported a difference in bacterial burden with SIGNR1 (*Cd209b*)-KO at one- and nine-months post infection, but by scoring Ziehl-Neelsen-stained lung sections rather than directly counting CFUs (140). When assessed, altered immunity was consistently observed with this KO. A single study found a difference in bacterial burden and immunological response with a SIGNR3 (*CD209d*)-KO but not a SIGNR5 (*Cd209a*)-KO (139). Another lone study addressing the mannose receptor showed no bacterial or immunologic effect with KO (140). Two studies on MINCLE presented opposing data on pulmonary bacterial burden (more or less a half log compared to WT) and only one identified significant immunological changes with KO. Double KO of the genes encoding mannose receptor plus SIGNR1 showed no bacterial or immunologic differences. KOs of other membrane-bound CLRs have not been tested during *in vivo Mtb* infection.

KOs of genes encoding collectins SP-A and SP-D had no long-term effect on bacterial burden during *Mtb* infection – survival was not tested/presented. Immunologic responses were similar to WT but with decreased neutrophil numbers in the lung. Double KO for SP-A and SP-D genes was similar to the SP-A single KO (145). KO of the gene encoding ficolin-A (homologue of human ficolin-2 and/or 3) decreased the survival of mice given one million CFU H37Rv strain i.v. compared to WT, but survival was enhanced compared to WT when KO mice were given a plasmid containing ficolin-A or ficolin-2 by i.m. electroporation on the day of infection (73). This suggests ficolins might help control systemic or bloodborne *Mtb*. Thus, as with TLRs, CLRs are generally dispensable for *Mtb* immunity. The few exceptions seem to suggest an early, minor role in *Mtb* infection for CLRs like MCL and SP-A/D.

### KOs of Certain Cytosolic PRRs Worsened *Mtb* Infection Outcome

NOD2 disruption produced a late survival phenotype: KO mice died faster than WT near 6 months post *Mtb* infection. Bacterial burden was slightly higher and immunological responses were also reduced in KO mice in this study (90). A separate study did not find significant differences in bacterial burden nor immunological responses with *Nod2*-KO nor *Nod2*-*Tlr2*-double KO, but survival was not evaluated (89). In contrast, *Nlrp3*-KO had no effect on bacterial burden in three studies. Immunological responses with *Nlrp3*-KO can be altered, but survival did not change.

*Aim2*-KO mice succumbed rapidly to infection with one million CFU H37Rv strain delivered i.t. compared to WT. The KO had greater bacterial burden and pathology and altered immunity (94). However, no other independent studies were found besides this one, and the high dose delivery makes the result difficult to compare to other PRR-KO survival studies with the more physiological low-dose aerosol infection. The importance of AIM2 during mycobacterial infection is supported by data with BCG, where repeated infection of WT and *Aim2*-KO mice vial the tail vein showed KO mice were defective in controlling bacterial burden which was associated with altered immunity (enhance type I IFN, reduced type II IFN) (101). Additionally, the adaptor protein ASC (a.k.a. PYCARD) was also shown to be important for survival in at least two separate studies (146, 155). This supports the importance of AIM2 and/or another inflammasome sensor for *Mtb* infection, with NLRP3 seemingly dispensable.

Pulmonary burden of *Mtb* in *Cgas*-KO and *Sting1* ^gt/gt^ mice was unchanged from WT at 3 and 6 weeks post aerosol infection of 200 CFU of the Erdman strain, although *Cgas*-KO mice had late reduced survival (deaths between 100 and 200 days p.i.) while the STING mutant did not differ from WT (97). In another study, an Erdman-strain aerosol experiment running 100 days did not reveal a difference between WT and *Cgas*-KO mice in terms of survival and bacterial burden, but less type I IFN was present in the lungs and serum (99). In a third study with i.n. infection with 1000 CFU H37Rv strain, cGAS and STING mutations did not affect survival past 250 days (no mice died as with WT), although *Cgas*-KO mice did not maintain weight as well. Bacterial burden and immunology were the same as WT too (151). Together, these studies suggest cGAS plays a minor role during *Mtb* infection (that emerged as a death phenotype late in one study), while STING is dispensable. These findings are difficult to reconcile with the proposed model where cGAS functions upstream of STING as the mycobacterial DNA sensor; accordingly a STING mutant should be defective for cGAS functions. Furthermore, *Mtb* CDC1551 mutants that either lack their own c-di-AMP production, or overexpress it, significantly decreased and increased survival relative to WT, respectively (100), contributing further confusion regarding the importance of STING. It is possible that the importance of cGAS during mouse survival of *Mtb* infection is related to a STING-independent function of cGAS, and that the STING phenotype with mutant *Mtb* is more valuable as a mechanistic lesson than a biologically relevant one.

### No Other PRR KOs Were Detrimental to the Host During *Mtb* Infection

No data was found showing scavenger receptor KOs were detrimental to *Mtb* control. A difference in bacterial burden during *Mtb* infection of *Marco*-KO mice was only detected by scoring Ziehl-Neelsen-stained lungs, not by CFU enumeration, at 6 and 9 months post infection (140). KOs for SR-A and SR-B genes did not result in increased bacterial burdens nor were they consistently associated with immunological changes. Two independent studies examining the complement receptor CR3/*Cd11b* KO during *Mtb* infection found no evidence that they play a role in *Mtb* control nor survival (153, 154).

In summary, most PRR KO experiments presented did not show reduced survival compared to WT. Control of bacterial burden was either unaffected or just slightly increased by PRR KOs in most experiments. We suspect that publication bias against negative data would also mean that PRR KO effects are, if anything, over-represented in the literature. In contrast, altered immunity was found often in PRR KOs during *Mtb* infection. It is possible that the effects on immunity with some PRR KOs are not large or relevant enough to result in changes in survival and bacterial burden that are sufficiently robust to be statistically detectable with a practical number of animals.

## PRR Diversity in Humans and Outcomes of *Mtb* Infection

Selective pressure caused by human-microbe interactions coupled with population admixture has helped shape the response of modern humans to pathogens (162). A recent example is a locus controlling COVID-19 severity in modern humans that can be traced to Neanderthal introgression (163). *Mtb* and humans have coexisted for an estimated 2,000 – 6,000 years (3, 4) and purifying selection of human genes by *Mtb* was traced to the bronze age for the *TYK2* P1104A mutation. Similarly, as members of the first line of host innate immune defense PRRs have been subjected to purifying selection (164). PRR diversity in humans may explain, at least in part, the variable susceptibility to *Mtb* across populations.

For example, humans express 10 functional TLRs which are subdivided in two categories: cell surface (TLR1, 2, 4 – 6 and 10) and intracellular endolysosomal (TLR3, 7 – 9). The intracellular TLRs underwent strong purifying selection and have poor tolerance to loss of function mutations (165). Conversely, cell surface TLRs are more permissive to genetic variation across human populations (166). This difference may be attributed to the nature of ligands. Bacterial antigens detected by cell surface TLRs are clearly distinct from host molecules while nucleic acids detected by intracellular TLRs (RNA or DNA with CpGs) can resemble host endogenous factors. It has been proposed that mutations in intracellular TLRs are less tolerated to prevent “autoimmunity” (167–169). *Mtb* is detected by heterodimers of TLR1, 2 and 6, therefore presenting redundancy in the host response. Interestingly, mutations in the *TLR1* (S248N, I602S), *TLR6* (P249S) and *TLR10* (I775V) genes clustered on chromosome 4p14 have shown signs of recent positive selection in Europeans (165). It has been suggested, although not confirmed, that tuberculosis and leprosy epidemics in Europe have played a role in this selective pressure (170). Of particular interest is the TLR1 I602S mutation which has been associated with both TB and leprosy (171–175). The TLR1 602S amino acid was shown to impair NF-κB activity in response to *Mtb* and decrease IL-6 production (174). Studies evaluating TLR2 mutations in TB have provided inconsistent results, which limited the interpretation of its role in TB pathogenesis (176, 177). Moreover, TLR4 and TLR9 have also been suggested to contribute to TB susceptibility (178–180).

NLR is another group of PRRs that shows signs of diversity between populations. NLRs encompass three families of cytosolic PRRs (NOD receptors, NLRPs and IPAFs) involved in viral and intracellular bacterial pathogen recognition. An excess of rare *NOD1* non-synonymous variants segregating in the human population provided evidence for weak negative selection against these variants (181). In contrast, there was evidence among Asians and Europeans of positive selection for rare variants in *NOD2* (181). In a meta-analysis, the NOD2 R702W amino acid change was associated with protection from TB (182, 183). Curiously, the same NOD2 R702W mutation is one of the strongest known genetic risk factors for Crohn’s disease, suggesting a pivotal role for NOD2 in balancing host inflammatory responses (184). Most NLRPs shows signs of strong selective constrains emphasizing their essential function in the human innate immune response (181). Macrophages challenged with *Mtb* or *M. marinum in vitro* showed a NLRP3-dependent increase in IL1*β* production (176). In a small population of cases with HIV/*Mtb* co-infection a non-coding variant in NLRP3 had a weak association with early mortality (185).

DC-SIGN (CD209), a member of CLR family, is a major dendritic cell receptor of *Mtb* (46). In ancient humans a duplication of *CD209* gave origin to the *CD209L* gene. Interestingly, natural selection has prevented accumulation of amino acid changes in CD209 while the closely related *CD209L* gene was permissive (186). This discrepancy in selective pressures highlights the importance of function for CD209 while diversity in CD209L might have benefitted human adaptation to pathogens. Two promoter variants in CD209 are associated with TB in multiple African populations (187–189), South Asians (190) and Brazilians (191). Other PRRs, such as ficolins, have been evaluated for association with TB (192, 193), while studies with genes encoding proteins of the complement and PRRs for the RIG-1 family have not yet been reported.

## How Some Non-PRR KOs Compare

Through animal experimentation, certain genes and associated pathways have been shown to be major determinants of the host outcome upon *Mtb* infection. Here, we define how some of these pathways compare to PRR pathways at the molecular level, and the level of importance to *Mtb* infection, as internal positive controls to our review.

For the sake of controls, similar systems to the PRR-MAMP interaction would include endogenous receptor-ligand systems. Receptor-cytokine interactions are an example which includes mechanisms that are even functionally related to PRR signalling pathways (e.g. the IL-1R/IL-1 system, which uses MyD88 like the TLRs as mentioned above).

IL-1R deficient mice were more susceptible to *Mtb* after intranasal infection with 10^5^ CFU H37Rv strain, with a median survival of around 110 days, while no WT had died by 140 days; the remaining KO mice had 4 logs more pulmonary CFU than WT at 140 days post infection (194). Another study by a different group showed that with 100 CFU Kurono strain aerosol infection *Il1r1* KO mice had died after 45 days (KO mice had 3 logs more pulmonary CFU than WT at 35 days) (195). During another H37Rv strain infection (200 CFU i.n.), *Il1r1* KO mice phenocopied *Myd88* KO mice (died around 4 weeks post infection) (159). There have been variable phenotypes with IL-1α and IL-1β deficiency: in one study *Il1b* KO was sufficient to phenocopy *Il1r1* KO (155); in another study the double cytokine KO only reduced pulmonary *Mtb* CFU control (196); in a third study only double cytokine KO, not single, shortened survival like *Il1r1* KO (197). Lastly, heterozygous deficiency of IL-1R antagonist, overexpressed in mice carrying the *Sst1* (super susceptibility to tuberculosis 1) locus, almost completely rescued these mice from their type-I IFN driven early mortality and excessive pulmonary CFU burden during *Mtb* Erdman strain infection, again highlighting the protective effect of IL-1R signalling (198). Thus, MyD88-dependent cytokine-receptor systems can be critical for *Mtb* control in mice.

In mice lacking TNF receptor, or treated with anti-TNF antibodies, mice succumbed to uncontrolled *Mtb* Erdman strain i.v. infection in about a month while WT controls all survived past 125 days (199). This result has been replicated in *Tnf* KO mice in numerous studies over the years (118, 160, 197). TNF receptor deficient mice died approximately as rapidly as *Tnf* KO, even if the receptor KO was only on myeloid cells; lymphoid cell receptor KO did not differ from WT (200). Thus, the TNF pathway is critical for *Mtb* control in mice to prevent rapid death. The importance of TNF with *Mtb* infection in humans was demonstrated when anti-TNF treatment was associated with the emergence of TB in patients receiving this treatment for other reasons (201).

Similarly, IFN-γ signalling has been known to be critical for control of *Mtb* in animal models for decades (202, 203). IFN-γ from CD4+ T cells in particular is necessary for survival, and animals lacking IFN-γ from just CD4+ T cells succumb after two months post aerosol infection; however IFN-γ’s role was mostly extrapulmonary with a limited role in the lungs (204). IL-12p40, upon which IFN-γ is partly dependent, has also been knocked-out in mice and resulted in uncontrolled replication of *Mtb* (Erdman strain, administered i.v.) and mortality within 1.5 months compared to WT mice which lived “to old age” (205). Human mutations in IFN-γ or IL-12 pathway genes causing impaired IFN-γ-mediated immunity result in Mendelian Susceptibility to Mycobacterial Disease, which manifests as childhood BCG dissemination or non-tuberculous mycobacterial infection, and occasionally *Mtb* infection later in those who live (206).

Cytokine and PRR signalling on their own do not have direct bactericidal effects – they are thought to modulate innate defense mechanisms and instruct adaptive immunity. The endgame of bacteriologic control are the host’s killing mechanisms, which in macrophages include low phagosomal pH, digestive enzymes like lysozymes, and reactive oxygen species. As an example, the well-studied nitric oxide is produced by NOS2 to attack *Mtb*. Mice lacking NOS2 all died within 50 days of i.v. infection with 10^5^ CFU Erdman strain while WT median survival was about 150 days (207). In a separate study, aerosol infection with 100 CFU H37Rv strain similarly resulted in death before day 50 associated with increased *Mtb* burden (208). Thus, effectors like nitric oxide are irreplaceable for control of *Mtb* and host survival.

## Why Have Genetic Studies of TB in Humans Been Underwhelming?

Genetic epidemiology studies have provided only a handful of PRR and non-PRR genes as global risk factors for TB. This lack of success is in striking contrast to leprosy, the second most common mycobacterial disease in humans (209). Strain diversity of *Mtb* compared to *M. leprae* might have played a role; however, the most likely cause for the lack of consistent results is phenotypic heterogeneity among TB cases. Most studies define TB as a single entity combining cases regardless of their clinical and biological characteristics. While this approach has worked for leprosy (210, 211), in other instances combining all leprosy cases has proven troublesome due to the presence of well-defined endophenotypes (212, 213). Common endophenotypes in leprosy are excessive host inflammatory responses, so-called lepra reactions, that sub-divide the overall group of patients. Endophenotypes can result in misclassification of genetic effects (213, 214). Indeed, the genetic associations can be in opposite direction between endophenotype and disease *per se* (212, 215).

Genetic modulators with opposing effects on unrecognized endophenotypes and clinically defined TB might be difficult to detect even in studies with very large sample sizes. This raises the question if similar, perhaps more complex endophenotypes, underlie the disappointing results from TB genetic studies. Specifically, considering the impact of PPR genes on intermediary immune phenotypes in the mouse, it is conceivable that PPR polymorphism may yet have a role to play in the genetics of TB pathogenesis. Heterogeneity among cases appears to be predominant in large scale genetic studies in TB and the existence of TB endotypes has been proposed (216). Recent advances in molecular and analytical techniques have allowed the identification of at least two TB endotypes through unbiased clustering of transcriptional changes in distinct molecular pathways (217). One endophenotype presented immune exhaustion resulting in poor prognosis compared to the second endophenotype.

What remains unclear is to what extent TB endophenotypes represent the continued progression of TB pathogenesis or if they are distinct forms of the same disease. More studies will be necessary to settle this question. Such future studies need to focus on defining endophenotypes with the full weight of omics approaches, keeping in mind that these better-resolution phenotypes may represent kinetic entities. Such a ‘systems-medicine’ definition of TB, in excess of clinical and microbiological data, is expected to improve power for efficient mapping of endophenotypes. Molecular (RNA, proteins and metabolites) and immune (cellular) phenotyping using blood can provide information for dissociating TB cases into endophenotypes. This is a two-step approach, where first identification of interindividual molecular/cellular similarities is done prior to the genetic study. How to deal with the genetic study in the second step would depend on the groups, but could be either a continuous phenotype or stratified by endophenotype. Using an omics signature would overcome the limitations where patients are clinically similar but the genetic cause of TB is not the same. Clinical heterogeneity with *Mtb* infection that is ambiguous (e.g. placement on a spectrum from TST positive to active TB) can be better-defined or bypassed with non-biased omics data. However, independently of the nature of TB endotypes, it is now clear that heterogeneity may impact on genetic studies of TB and perhaps shed new light on the role of PPR polymorphisms.

## Final Thoughts and Conclusion

PRRs appear to be important for immunologic responses but have a more subtle role in control of *Mtb* and the course of TB. We hypothesize that this is partly due to the redundancy of many PRRs sensing different *Mtb* MAMPs. Amongst this redundancy, however, there may be unique immunological adjustments performed by specific PRRs. In contrast, genes that produce products mediating distinct effects, like IFN-*γ*, IL-12, nitric oxide and TNF are clearly essential to the host’s survival. Although we can consider PRRs ‘less important’ than effectors, this prompts an interesting question: Is this a situation of reduced selective pressure, which explains human PRR diversity? It is imaginable that the immunological outcome performed by an orchestra of PRRs can be quite varied as individual PRR activities are tuned differently by genetics. By contrast, altering the potency of an effector like IFN-γ would directly correlate with *Mtb* control, and therefore selection would be purifying.

Human genetic association studies of TB have yielded but a few promising leads. Animal and cellular human data clearly demonstrate that PRRs affect immunity during *Mtb* infection, despite small and/or delayed survival and bacteriologic phenotypes in PRR KO mice. Thus, PRR mutation in humans might manifest in endophenotypes of *Mtb* infection – states of altered immunity wherein the progression of TB may possess subtly different parameters. Defining such endophenotypes of *Mtb* infection through molecular and immunological profiling of patients may provide a roadmap on which to trace the effects of PRR variation on the course of TB.

## Data collection, processing and presentation

Literature searches were performed on the Medline database with Pubmed and results were collected and curated using Endnote X9 (Clarivate Analytics, USA). The text, table and figure were created with Microsoft Word, Excel and PowerPoint, respectively.

## Author Contributions

All authors listed have made a substantial, direct, and intellectual contribution to the work, and approved it for publication.

## Funding

J-YD is supported by a Canadian Institutes of Health Research (CIHR) Canada Graduate Scholarship – Master’s Program, Fonds de Recherche du Québec – Santé (FRQ-S) Doctoral Training Award, RI-MUHC studentships and scholarships from the McGill Department of Microbiology and Immunology. MB: CIHR foundation grant (FDN-148362). ES CIHR foundation grant (FDN-143332) grant by NIH (1R01AI124349).

## Conflict of Interest

The authors declare that the research was conducted in the absence of any commercial or financial relationships that could be construed as a potential conflict of interest.

## Abbreviations

BCG, Bacille Calmette-Guérin, attenuated *M. bovis* vaccine against *Mtb*; CFUs, Colony-forming units, a measure of the amount of bacteria; CLR, C-type lectin receptor; Collectins, Soluble CLR; KO, Knockout, removal of a gene from a genome; LAM, Lipoarabinomannan, mannosylated = ManLAM, uncapped = araLAM; LPS, Lipopolysaccharide, a.k.a. endotoxin, MAMP from Gram negative bacteria; MAMP, Microbe-associated molecular pattern, recognized by host PRR(s); MDP, Muramyl dipeptide; MHC, Major histocompatibility complex; *Mtb, Mycobacterium tuberculosis*, causative agent of TB; NLR, NOD-like receptor; NOD, Nucleotide-binding oligomerization domain-containing; PIMs, phosphatidylinositol mannosides; PRR, Pattern recognition receptor, recognize MAMP(s); TB, Tuberculosis, the disease caused by *Mtb*; TDM, Trehalose dimycolate; TLR, Toll-like receptor; TST, Tuberculin skin test, indicates adaptive T-cell response to mycobacterial antigens.
